# White-Nose Syndrome Fungus in a 1918 Bat Specimen from France 

**DOI:** 10.3201/eid2309.170875

**Published:** 2017-09

**Authors:** Michael G. Campana, Naoko P. Kurata, Jeffrey T. Foster, Lauren E. Helgen, DeeAnn M. Reeder, Robert C. Fleischer, Kristofer M. Helgen

**Affiliations:** Smithsonian Institution, Washington, DC, USA (M.G. Campana, N.P. Kurata, L.E. Helgen, R.C. Fleischer);; University of New Hampshire, Durham, New Hampshire, USA (J.T. Foster);; Bucknell University, Lewisburg, Pennsylvania, USA (D.M. Reeder);; University of Adelaide, Adelaide, South Australia, Australia (K.M. Helgen)

**Keywords:** White-nose syndrome, Pseudogymnoascus destructans, bats, Myotis bechsteinii, France, fungi

## Abstract

White-nose syndrome, first diagnosed in North America in 2006, causes mass deaths among bats in North America. We found the causative fungus, *Pseudogymnoascus* *destructans*, in a 1918 sample collected in Europe, where bats have now adapted to the fungus. These results are consistent with a Eurasian origin of the pathogen.

We report the earliest known historical incidence of the fungus *Pseudogymnoascus* (formerly *Geomyces*) *destructans*, detected in a museum specimen of a bat (*Myotis bechsteinii*) collected in France in 1918. This fungal pathogen causes white-nose syndrome (WNS) in bats (*1*). Since its introduction into eastern North America around 2006, WNS has devastated bat populations across the continent (*2*). *P. destructans* has also been found across the Eurasian landmass (*3**,**4*) without documented mass bat deaths. Epidemiologic evidence among bats and fungal genetics indicate that the fungus has been recently introduced into North American bat populations (*5**–**7*).

To clarify the epidemiologic history of WNS and to investigate physical evidence of its presence in specific locations in the past, we screened 138 19th- and 20th-century bat specimens (housed at the National Museum of Natural History [USNM], Washington, DC) from North America (n = 41), Europe (n = 83), and East Asia (n = 14) for *P. destructans* DNA (Technical Appendix). We sampled dry museum skins and intact bodies stored in 70% ethanol; some were originally fixed in formalin. We swabbed bat rostra and wings to collect potentially preserved *P. destructans* biomolecules and stored swabs in 100% ethanol until DNA extraction.

We extracted DNA in a dedicated ancient DNA laboratory at the National Zoological Park (Washington, DC) by using stringent protocols to prevent false positive results from modern DNA contamination (*8*). Before extraction, we removed swabs from the ethanol and let them air dry. We then let swabs digest overnight at 55°C in 600 μL extraction buffer (1× Tris-EDTA buffer, pH 8.0, 0.019 mmol/L EDTA, 0.01 mmol/L NaCl, 1% SDS, 10 mg/mL DTT, and 1 mg/mL proteinase K) (*8*). Later extractions omitted DTT. We extracted digested samples twice in 600 μL phenol and once in 600 μL chloroform. We removed and concentrated the aqueous phase by using Amicon Ultra-4 30 kDA molecular weight cutoff columns (Millipore Sigma, Merck, Billerica, MA, USA) to a final volume of ≈250 μL. We included 1 extraction blank for every 10–11 historical samples. 

We screened extracts for *P. destructans* by using a previously described species-specific quantitative PCR targeting 103 bp (including primers) of the intergenic spacer region (*9*). Each extract was amplified in 2–8 replicate PCRs. Multiple, no-template controls (*2*,*3*) were included in each PCR setup. Positive products from experiments in which quantifiable contamination (>0.1 genome equivalents/μL sample) was observed in >1 negative control were discarded; these experiments were repeated with fresh reagents.

One sample (USNM 231170) tested positive in 2 of 3 PCRs. We performed a second independent extraction on this sample. The replicate extraction tested positive in 4 of 5 PCRs. Two of the USNM 231170–positive PCR products were confirmed by using Sanger sequencing and comparison to publicly available *P. destructans* sequences in GenBank. These sequences were 100% identical to *P. destructans* sequences from North America (GenBank accession nos. JX270192.1 and JX415267.1). In addition, 2 samples (USNM 15513 and 154222) yielded positive products in a single PCR each, but we were unable to replicate these results. No usable sequence was obtained from the USNM 15513 amplicon; the USNM 154222 sequence differed from the North American sequence by a single thymine deletion. DNA sequences were deposited in GenBank (accession nos. MF370925–6).

The 1 confirmed case of *P. destructans* infection among the museum samples we studied (USNM 231170, male, skin and skull) was in a Bechstein’s bat (*Myotis bechsteinii*) collected on May 9, 1918, at Forêt de Russy, Centre-Val de Loire, France. This sequence is unlikely to represent *P. destructans* from a recently collected infected bat from North America because recently collected specimens have been purposefully stored with care in a separate room within the USNM mammal department, away from the historical bat collection. Furthermore, none of the historical samples of bats collected in North America, which are more likely to be cross-contaminated with potentially infected specimens compared with European specimens, tested positive for *P. destructans.*

We provide evidence of the presence of *P. destructans* ≈100 years ago in Europe. In addition, we found no evidence of *P. destructans* in bats collected in eastern North America during 1861–1971. Although false negatives are highly likely because of the age, preparation, and storage of these specimens, these results are consistent with a Eurasian origin of the current WNS epidemic and strong association of the fungus with Eurasian bats of the genus *Myotis* (*3**,**6**,**10*). Bats across Eurasia have adapted to *P*. *destructans* over more than a century, but the fungus was initially detected in North America during the early 21st century, and the bats on this continent have no immunity. This result extends the documented temporal occurrence of *P. destructans* as a bat-associated fungus to the early 20th century and highlights the value of archived museum specimens for epidemiologic study of emerging fungal diseases, including WNS.

Technical Appendix Results of screening 138 historic bat specimens for *Pseudogymnoascus destructans*, the fungus associated with white-nose syndrome.
