# Supplementary material for: White-Nose Syndrome Fungus in a 1918 Bat Specimen from France
Source: Emerg Infect Dis. 2017 Sep;23(9):1611–2. doi: 10.3201/eid2309.170875 (PMC5572869; doi:10.3201/eid2309.170875)
Supplement: Technical Appendix — Results of screening 138 historic bat specimens for Pseudogymnoascus destructans, the fungus associated with white-nose syndrome. [file 17-0875-Techapp-s1.pdf]

Article DOI: https://doi.org/10.3201/eid2309.17075

White-Nose Syndrome Fungus in a 1918 Bat Specimen from France

Technical Appendix

Technical Appendix Table. Bat specimens screened for *Pseudogymnoascus destructans*

USNM no.	Species	Region	Collection location	Collection date	Preparation
11189	*Myotis brandtii*	East Asia	Petropavlovsk-Kamchatsky, Kamchatka Krai, Russia	Pre-1900	Fluid
155523	*Myotis brandtii*	East Asia	Petropavlovsk-Kamchatsky, Kamchatka Krai, Russia	1889 Jul	Fluid
240190	*Myotis chinensis*	East Asia	Changsha, Hunan, China	1923 Jul 26	Skin
240191	*Myotis chinensis*	East Asia	Changsha, Hunan, China	1923 Jul 26	Skin
240215	*Myotis chinensis*	East Asia	Changsha, Hunan, China	1923 Jul 26	Fluid
240216	*Myotis chinensis*	East Asia	Changsha, Hunan, China	1923 Jul 26	Fluid
240217	*Myotis chinensis*	East Asia	Changsha, Hunan, China	1923 Jul 26	Fluid
253368	*Myotis daubentonii*	East Asia	Sichuan, China	1920s	Fluid
279358	*Myotis altarium*	East Asia	Guìyáng, Guizhou, China	1945 Aug 26	Skin
279359	*Myotis altarium*	East Asia	Guìyáng, Guizhou, China	1945 Aug 26	Skin
279360	*Myotis altarium*	East Asia	Guìyáng, Guizhou, China	1945 Sep 1	Skin
279361	*Myotis altarium*	East Asia	Guìyáng, Guizhou, China	1945 Sep 1	Skin
356343	*Myotis brandtii*	East Asia	Seoul, South Korea	1952 Aug 11	Fluid
302908	*Myotis daubentonii*	East Asia	Daegu, Yeongnam, South Korea	1955 Jun 15	Fluid
5569	*Myotis mystacinus*	Europe	Bavaria, Germany	Pre-1862	Skin
5570	*Myotis mystacinus*	Europe	Bavaria, Germany	Pre-1862	Skin
12335	*Myotis daubentonii*	Europe	Gotthard, Ticino, Switzerland	Pre-1900	Skin
13516	*Myotis mystacinus*	Europe	Dresden, Saxony, Germany	1873 Jun 10	Skin
14535	*Rhinolophus euryale*	Europe	Saint-Paterne, Pays-de-la-Loire, France	Pre-1884	Fluid
15512	*Myotis mystacinus*	Europe	Skåne County, Götaland, Sweden	1883 Mar	Fluid
15603	*Rhinolophus ferrumequinum*	Europe	France	Pre-1900	Skin
15604	*Rhinolophus hipposideros*	Europe	France	Pre-1900	Skin
15605	*Myotis nattereri*	Europe	France	Pre-1900	Skin
15606	*Myotis mystacinus*	Europe	France	Pre-1900	Skin
15607	*Plecotus auritus*	Europe	France	Pre-1900	Skin
18523	*Myotis capaccinii*	Europe	Marseilles, Provence-Alpes-Côte D'Azur, France	Pre-1900	Fluid
18528	*Myotis mystacinus*	Europe	South Tyrol, Italy	Pre-1900	Fluid
85182	*Myotis daubentonii*	Europe	Uppsala, Uppsala, Sweden	1898 Aug	Fluid
85519	*Myotis mystacinus*	Europe	Belgium	Pre-1900	Skin
86574	*Myotis daubentonii*	Europe	Lecco, Lombardy, Italy	1898 Feb	Fluid
86926	*Eptesicus serotinus*	Europe	Barsac, Aquitaine-Limousin-Poitou-Charentes, France	1897 Mar 28	Fluid
101326	*Myotis myotis*	Europe	Cadillac, Aquitaine-Limousin-Poitou-Charentes, France	1899 Jun	Skin
102468	*Rhinolophus hipposideros*	Europe	Marseilles, Provence-Alpes-Côte D'Azur, France	1900 Jun	Fluid
102469	*Rhinolophus ferrumequinum*	Europe	Marseilles, Provence-Alpes-Côte D'Azur, France	Received 1900 Jun	Fluid
102470	*Myotis myotis*	Europe	Marseilles, Provence-Alpes-Côte D'Azur, France	Received 1900 Jun	Fluid
102500	*Pipistrellus pipistrellus*	Europe	Marseilles, Provence-Alpes-Côte D'Azur, France	Received 1900 Jun	Fluid
102501	*Pipistrellus kuhlii*	Europe	Marseilles, Provence-Alpes-Côte D'Azur, France	Received 1900 Jun	Fluid
102502	*Miniopterus schreibersii*	Europe	Marseilles, Provence-Alpes-Côte D'Azur, France	Received 1900 Jun	Fluid
102503	*Plecotus auritus*	Europe	Marseilles, Provence-Alpes-Côte D'Azur, France	Received 1900 Jun	Fluid

USNM no.	Species	Region	Collection location	Collection date	Preparation
113855	*Myotis myotis*	Europe	Tagerwilen, Thurgau, Switzerland	1900 Jun 9	Fluid
113858	*Myotis myotis*	Europe	Tagerwilen, Thurgau, Switzerland	1900 Jun 9	Fluid
113861	*Myotis myotis*	Europe	Tagerwilen, Thurgau, Switzerland	1900 Jun 9	Fluid
113866	*Myotis nattereri*	Europe	Thurgau, Switzerland	1900 Jun 9	Fluid
113868	*Myotis nattereri*	Europe	Thurgau, Switzerland	1900 Jun 9	Fluid
120994	*Myotis myotis*	Europe	Lipová, Ústi nad Labem, Czech Republic	1902 Sep 24	Skin
120995	*Myotis myotis*	Europe	Lipová, Ústi nad Labem, Czech Republic	1902 Sep 24	Skin
120998	*Myotis nattereri*	Europe	Moritzburg, Saxony, Germany	1902 Nov 5	Skin
120999	*Myotis nattereri*	Europe	Moritzburg, Saxony, Germany	1902 Nov 5	Skin
121000	*Myotis nattereri*	Europe	Moritzburg, Saxony, Germany	1902 Nov 5	Skin
121191	*Myotis myotis*	Europe	Andermatt, Uri, Switzerland	1902 Aug 20	Skin
152524	*Rhinolophus ferrumequinum*	Europe	Dions, Languedoc-Rousillon-Midi-Pyrénées, France	1907 Oct 10	Skin
152525	*Rhinolophus ferrumequinum*	Europe	Troubate, Pyrenees, France	1900 Jan 14	Skin
152526	*Rhinolophus ferrumequinum*	Europe	Troubate, Pyrenees, France	900 Jan 20	Skin
152528	*Rhinolophus hipposideros*	Europe	Dions, Languedoc-Rousillon-Midi-Pyrénées, France	1907 Oct 11	Skin
152529	*Rhinolophus hipposideros*	Europe	Dions, Languedoc-Rousillon-Midi-Pyrénées, France	1907 Oct 11	Skin
152533	*Rhinolophus euryale*	Europe	Grotte de Meounes, Provence-Alpes-Côte D'Azur, France	1908 Jan	Fluid
152534	*Rhinolophus euryale*	Europe	Grotte de Meounes, Provence-Alpes-Côte D'Azur, France	1908 Jan	Fluid
152535	*Rhinolophus euryale*	Europe	Grotte de Meounes, Provence-Alpes-Côte D'Azur, France	1908 Jan	Fluid
152539	*Myotis daubentonii*	Europe	Uppsala, Uppsala, Sweden	1895 May 21	Skin
152556	*Pipistrellus pipistrellus*	Europe	Santo Domingo de Silos, Castile and León, Spain	Pre-1910	Fluid
152600	*Miniopterus schreibersii*	Europe	Dions, Languedoc-Rousillon-Midi-Pyrénées, France	1907 Oct 10	Skin
152601	*Miniopterus schreibersii*	Europe	Dions, Languedoc-Rousillon-Midi-Pyrénées, France	1907 Oct 10	Skin
152602	*Miniopterus schreibersii*	Europe	Dions, Languedoc-Rousillon-Midi-Pyrénées, France	1907 Oct 10	Skin
152603	*Miniopterus schreibersii*	Europe	Dions, Languedoc-Rousillon-Midi-Pyrénées, France	1907 Oct 10	Skin
152604	*Miniopterus schreibersii*	Europe	Dions, Languedoc-Rousillon-Midi-Pyrénées, France	1907 Oct 10	Skin
152605	*Miniopterus schreibersii*	Europe	Dions, Languedoc-Rousillon-Midi-Pyrénées, France	1907 Oct 10	Skin
152606	*Miniopterus schreibersii*	Europe	Dions, Languedoc-Rousillon-Midi-Pyrénées, France	1907 Oct 10	Skin
152607	*Miniopterus schreibersii*	Europe	Dions, Languedoc-Rousillon-Midi-Pyrénées, France	1907 Oct 10	Skin
154080	*Myotis bechsteinii*	Europe	Hungary	Pre-1910	Fluid
154081	*Myotis bechsteinii*	Europe	Hungary	Pre-1910	Fluid
154221	*Rhinolophus ferrumequinum*	Europe	Nîmes, Languedoc-Rousillon-Midi-Pyrénées, France	Pre-1910	Skin
154222†	*Rhinolophus euryale*	Europe	Gard Department, Languedoc-Rousillon-Midi-Pyrénées, France	Pre-1910	Skin
172121	*Rhinolophus hipposideros*	Europe	Ax-les-Thermes, Languedoc-Rousillon-Midi-Pyrénées, France	1906 Aug 18	Skin
172127	*Myotis oxygnathus*	Europe	Near Burgos, Castile and León, Spain	1906 Oct 3	Skin
172128	*Pipistrellus kuhlii*	Europe	Saint-Gilles, Languedoc-Rousillon-Midi-Pyrénées, France	1908 May	Skin
172304	*Rhinolophus hipposideros*	Europe	Ax-les-Thermes, Languedoc-Rousillon-Midi-Pyrénées, France	1906 Oct 18	Fluid
187431	*Myotis daubentonii*	Europe	Christchurch, Isle of Wight, UK	Pre-1910	Fluid
231169	*Myotis bechsteinii*	Europe	Forêt de Russy, Centre-Val de Loire, France	1918 May 3	Skin
231170*	*Myotis bechsteinii*	Europe	Forêt de Russy, Centre-Val de Loire, France	1918 May 9	Skin
231171	*Myotis bechsteinii*	Europe	Forêt de Russy, Centre-Val de Loire, France	1918 May 9	Skin
260651	*Pipistrellus pipistrellus*	Europe	Madrid, Madrid, Spain	1934 Mar 31	Skin
260656	*Myotis myotis*	Europe	Riofrío, Castile and León, Spain	1933 May 2	Skin
260657	*Myotis myotis*	Europe	Riofrío, Castile and León, Spain	1933 Feb 5	Skin
260659	*Myotis myotis*	Europe	Almadenejos, Castile-La Mancha, Spain	1930s	Fluid
260660	*Myotis myotis*	Europe	Almadenejos, Castile-La Mancha, Spain	1930s	Fluid

USNM no.	Species	Region	Collection location	Collection date	Preparation
260663	*Miniopterus schreibersii*	Europe	Pyrénées-Orientales, Languedoc-Rousillon-Midi-Pyrénées, France	1930s	Fluid
260664	*Miniopterus schreibersii*	Europe	Pyrénées-Orientales, Languedoc-Rousillon-Midi-Pyrénées, France	1930s	Fluid
260665	*Miniopterus schreibersii*	Europe	Pyrénées-Orientales, Languedoc-Rousillon-Midi-Pyrénées, France	1930s	Fluid
260673	*Pipistrellus pipistrellus*	Europe	Ruidera, Castilla-La Mancha, Spain	1934 Jul 11	Fluid
303346	*Plecotus austriacus*	Europe	Saint-Vaast-la-Hougue, Normandy, France	1956 Aug 21	Skin
345233	*Myotis myotis*	Europe	Augsburg, Bavaria, Germany	1957 Aug 1	Fluid
345234	*Myotis myotis*	Europe	Augsburg, Bavaria, Germany	1960 Jul 17	Fluid
345235	*Myotis daubentonii*	Europe	Bavarian Forest, Bodenwöhr, Bavaria, Germany	1959 May 9	Fluid
540784	*Myotis mystacinus*	Europe	"Izbica" Cave, Slovakia	1961 Feb 12	Skin
12337	*Myotis myotis*	Europe	Gotthard, Ticino, Switzerland	Pre-1900	Skin
15513†	*Myotis bechsteinii*	Europe	Ronnemollo, Sweden	1883 Mar 5	Fluid
85518	*Myotis mystacinus*	Europe	Belgium	Pre-1900	Skin
5334	*Myotis lucifugus*	North America	West Point, New York, USA	Pre-1861	Fluid
22228	*Myotis sodalis*	North America	Centre County, Pennsylvania, USA	1892 Feb 7	Fluid
71926	*Myotis leibii*	North America	Sing Sing, New York, USA	1884 Jun 9	Fluid
84888	*Myotis septentrionalis*	North America	Markleton, Pennsylvania, USA	1898 Aug 12	Skin
96957	*Myotis septentrionalis*	North America	Wilmington, Massachusetts, USA	1898 Oct 1	Skin
114015	*Myotis septentrionalis*	North America	Plummer’s Island, Maryland, USA	1902 Jun 1	Skin
117109	*Myotis septentrionalis*	North America	Plummer’s Island, Maryland, USA	1902 Mar 31	Skin
140765	*Myotis septentrionalis*	North America	Peterboro, New York, USA	1900 Sep 11	Skin
187295	*Myotis lucifugus*	North America	Howes Cave, Schoharie County, New York, USA	1884 Nov 18	Fluid
187300	*Myotis lucifugus*	North America	Howes Cave, Schoharie County, New York, USA	1884 Nov 18	Fluid
187358	*Myotis lucifugus*	North America	Keene Valley, Essex County, New York, USA	1880 Jul	Skin
187853	*Myotis leibii*	North America	Sing Sing, New York, USA	1881 Jun 29	Fluid
205961	*Myotis lucifugus*	North America	Proctor, Vermont, USA	1915 Jan 11	Skin
206577	*Myotis sodalis*	North America	Proctor, Vermont, USA	1915 Apr 2	Skin
206580	*Myotis sodalis*	North America	Proctor, Vermont, USA	1915 Apr 2	Skin
206801	*Myotis lucifugus*	North America	Proctor, Vermont, USA	1915 May 4	Skin
249133	*Myotis grisescens*	North America	Dixon Cave, Mammoth Cave National Park, Kentucky, USA	1929 Jul 6	Skin
249138	*Myotis leibii*	North America	Crystal Cave, Mammoth Cave National Park, Kentucky, USA	1929 Aug 22	Skin
249139	*Myotis leibii*	North America	Mammoth Cave National Park, Kentucky, USA	1929 Aug 22	Skin
252560	*Myotis grisescens*	North America	Marvel Cave, Stone County, Missouri, USA	1927 Sep 27	Fluid
268446	*Myotis lucifugus*	North America	Laurel Caverns, Fayette County, Pennsylvania, USA	1938 Oct	Fluid
270287	*Myotis lucifugus*	North America	Dulany’s Cave, Uniontown, Pennsylvania, USA	1941 Apr 20	Skin
270288	*Myotis lucifugus*	North America	Dulany’s Cave, Uniontown, Pennsylvania, USA	1941 Apr 20	Skin
270289	*Myotis lucifugus*	North America	Dulany’s Cave, Uniontown, Pennsylvania, USA	1941 Apr 20	Skin
282666	*Myotis lucifugus*	North America	Pecks Lake, Pike County, Pennsylvania, USA	1947 Jul 25	Fluid
282770	*Myotis lucifugus*	North America	Deposit, New York, USA	1947 Aug 11	Skin

USNM no.	Species	Region	Collection location	Collection date	Preparation
282771	*Myotis lucifugus*	North America	Deposit, New York, USA	1947 Aug 11	Skin
282772	*Myotis lucifugus*	North America	Little Broken Straw Creek, Warren County, Pennsylvania, USA	1947 Aug 19	Fluid
288567	*Myotis lucifugus*	North America	Aeolus Cave, East Dorset, Vermont, USA	1962 Jul 15	Skin
288982	*Myotis lucifugus*	North America	Bearton Reservation, Monterey, Massachusetts, USA	1963 Jul 19	Skin
288984	*Myotis lucifugus*	North America	Bearton Reservation, Monterey, Massachusetts, USA	1963 Jul 19	Skin
288986	*Myotis lucifugus*	North America	Winchendon, Massachusetts, USA	1963 Aug 6	Skin
296704	*Myotis leibii*	North America	Near Hyndford, Renfrew County, Ontario, Canada	1942 Apr 4	Skin
296705	*Myotis leibii*	North America	Near Hyndford, Renfrew County, Ontario, Canada	1942 Apr 4	Skin
314985	*Myotis leibii*	North America	Breathing Cave, Burnsville, Virginia, USA	1960 Dec 11	Skin
349702	*Myotis lucifugus*	North America	Piscataway Park, Accokeek, Maryland, USA	1971 Aug 20	Skin
505449	*Myotis lucifugus*	North America	Rohrersville Cave #3, Locust Grove, Maryland, USA	1970 Oct 24	Skin
505455	*Myotis lucifugus*	North America	John Friends Cave, Garrett County, Maryland, USA	1971 Feb 27	Skin
526781	*Myotis septentrionalis*	North America	Wilmington, Massachusetts, USA	1899 Sep 9	Skin
32317	*Myotis sodalis*	North America	Centre County, Pennsylvania, USA	1892 Feb 11	Fluid
364632	*Myotis leibii*	North America	Tawney's Cave, Giles County, Virginia, USA	1964 Aug 17	Skin

**P. destructans*-positive specimen.
†Samples that yielded non-replicated positive PCR products.
